# Identifying and Evaluating Patient-Centered Mobile and Web Apps for Patients With Chronic Spontaneous Urticaria: Systematic Search and Content Analysis

**DOI:** 10.2196/71435

**Published:** 2026-03-02

**Authors:** Caroline Glatzel, Bernadette Glatzel, Tassilo Dege, Lena Brückel, Matthias Goebeler, Astrid Schmieder

**Affiliations:** 1 Department of Dermatology, Venereology and Allergology, University Hospital Würzburg, Josef-Schneider-Straße 2, Würzburg, D - 97080, Germany, +49 93120126369

**Keywords:** urticaria, mobile health apps, MHA, mobile apps, Mobile Application Rating Scale, MARS, user version of MARS, uMARS, German mHealth App Usability Questionnaire, G-MAUQ, Mobile Device Proficiency Questionnaire, MDPQ-16, affinity for technology interaction, ATI, skin

## Abstract

**Background:**

Chronic spontaneous urticaria (CSU) is characterized by recurrent wheals or angioedema lasting for more than 6 weeks and substantially affecting the quality of life. Given its fluctuating course, accurate symptom monitoring is essential. Mobile health apps (MHAs) offer promising tools for real-time symptom tracking, patient education, and communication. Systematic evaluation of existing MHAs for CSU is critical to inform the development of effective, patient-centered digital solutions.

**Objective:**

This study aimed to identify and evaluate publicly available MHAs for patients with CSU, assessing their quality, usability, and alignment with the needs of both patients and physicians to guide the development of future patient-centered apps.

**Methods:**

A systematic search of app stores and the internet was conducted to identify MHAs for CSU. Inclusion required German or English language support and patient-centered content. Apps were excluded if they contained advertisements; lacked patient-centered content, designed to assist patients in the self-management and care of their condition; or were focused on clinical trials or health care professional use. After screening, 1 app, CRUSE Control, met all criteria and was evaluated by 23 physicians and 16 patients with CSU using the German versions of the Mobile Application Rating Scale (MARS and end-user version of MARS) and the mHealth App Usability Questionnaire. Participants’ technical affinity was assessed using the affinity for technology interaction scale and the Mobile Device Proficiency Questionnaire. Additionally, they completed a custom questionnaire on their personal needs and expectations for CSU-specific MHAs.

**Results:**

Fifteen MHAs were identified, with 12 available on both platforms. Eleven apps were excluded due to lack of specificity to CSU (n=10) or not being patient-centered (n=1). One app, CRUSE Control, met all inclusion criteria and was selected for final evaluation. CRUSE Control received similar mean (SD) quality ratings from physicians (MARS 4.03, SD 0.45) and patients (end-user version of MARS: 4.06, SD 0.40; *P*=.83). Among the MARS subcategories, functionality was rated significantly higher by patients than by physicians (4.75, SD 0.41 vs 4.47, SD 0.55; *P*=.04). Usability, measured using the German mHealth App Usability Questionnaire (assessing effectiveness, efficiency, and satisfaction), showed no significant difference between physicians (5.85, SD 0.71) and patients (5.76, SD 0.41; *P*=.64). Technology affinity was comparable between groups, with physicians scoring 3.50 (SD 0.66) and patients 4.00 (SD 0.88) on the affinity for technology interaction (*P*=.05). Proficiency with mobile devices, assessed via the Mobile Device Proficiency Questionnaire, also showed similar results (physicians: 4.81, SD 0.26; patients: 4.74, SD 0.45; *P*=.60).

**Conclusions:**

Few high-quality MHAs for CSU are currently available, and only 1 met the inclusion criteria. Patients and physicians rated the app highly, though patients placed greater emphasis on functionality. High technology affinity in both groups supports adoption. Patients prioritized features that facilitate disease management. Although limited to a single app, these findings suggest that MHAs may support CSU care.

## Introduction

### Background

Urticaria is a common skin disorder characterized by the sudden onset of wheals and/or angioedema. Wheals (urticaria) typically presents with three key features: (1) sharply demarcated, superficial swellings of the skin of variable size and shape, usually surrounded by a reflex erythema; (2) intense pruritus or, less commonly, a burning sensation; and (3) a transient course, with lesions usually resolving within 30 minutes to 24 hours.

In around 40% of cases, episodes of urticaria are accompanied by angioedema, primarily affecting the face, but also the hands and feet [1]. Angioedema is defined by (1) rapid-onset, pronounced swelling in the deeper dermis, subcutis, or mucous membranes, often erythematous or skin-colored; (2) a sensation of tingling, burning, or tension and sometimes pain rather than itch; and (3) a slower resolution, typically lasting 72 hours. According to current guidelines, urticaria is classified by duration as either acute (≤6 wk) or chronic (>6 wk) and by trigger as spontaneous (no identifiable cause) or inducible (triggered by specific stimuli) [2,3].

The lifetime prevalence of urticaria is estimated to range between 10% to 20%, indicating that a substantial proportion of the population experiences at least one episode during their lifetime [2,4]. Chronic urticaria (CU) has a lifetime prevalence of approximately 1% to 2% among adults in Europe, the United States, and worldwide, with chronic spontaneous urticaria (CSU) being the most common subtype [4-6]. It most frequently manifests between the third and fifth decades of life [5,7,8] and affects women twice as often as men [8].

The impact of CSU on patients and their social environment is substantial and far-reaching [9]. Quality of life is markedly impaired, comparable to other severe dermatological conditions such as hidradenitis suppurativa or advanced atopic dermatitis [10]. The unpredictability of symptoms—such as itching, swelling, and wheals, which can lead to sleep disorders—contributes to emotional distress, particularly anxiety and depression [11-14].

CSU is primarily a clinical diagnosis, typically first assessed by general practitioners. A comprehensive diagnostic evaluation includes a detailed medical history, structured around the “7 C’s” (Confirm, Cause, Cofactors, Comorbidities, Consequences, Components, and Course), as well as the assessment of disease activity, impact on patient’s life, and disease management [2,15]. CU may also be triggered by circulating immune complexes and activation of the complement system. In cases persisting for several months or longer, autoimmune thyroiditis should be ruled out [16,17]. Standardized tools such as the urticaria activity score (UAS), Urticaria Control Test (UCT), and the Chronic Urticaria Quality of Life Questionnaire are recommended for quantifying disease burden and monitoring fluctuations over time [2,18].

Due to the episodic nature of CSU, which may persist for months or even years [19], symptom fluctuation is common and can make it difficult for health care providers to fully assess the disease’s burden.

To bridge this gap, mobile technologies have gained traction in dermatology. Clinical photography has been used for documenting disease progression and supporting diagnostic accuracy [20], and messaging tools and teleconsultation already support remote assessment and communication [21]. This offers additional benefits in improving treatment monitoring and enhancing patient education [22]. A recent study has demonstrated the feasibility and high acceptance of teledermatologic disease management in a cohort of patients with CSU [23]. Building on these existing digital tools, mobile health apps (MHAs) offer promising opportunities for the management of CSU. As the demand for accessible and flexible health care grows, they are becoming increasingly relevant in disease management, enabling real-time symptom documentation, remote monitoring, and timely interventions [24-27]. Their portability, good availability, and widespread use make MHAs particularly valuable for managing health issues, as reflected in the growing digital health market. By the third quarter of 2020, the Apple App Store offered 48,608 medical mHealth apps (up from 44,384 in Q3 2019) and a total of 82,633 health and fitness apps [28].

In Germany, MHAs can be certified as digital health apps (DiGAs), making them eligible for prescription and reimbursement through public health insurance since 2019 [29,30]. As part of the Digital Healthcare Act—the first law worldwide to integrate digital therapeutics into public care—DiGAs support patient self-management, improve therapy adherence, and facilitate communication by enabling the sharing of patient-generated data [29].

Challenges such as reimbursement issues, digital literacy, and variable acceptance among health care providers and patients limit the use of DiGAs, and currently, no certified DiGA or high-quality app exists specifically for CSU [31,32]. Given the clinical need and patient demand, identifying and evaluating existing MHAs for CSU is essential to guide the development of effective, user-centered digital solutions.

### Objective

The aim of this study was to identify MHAs currently available for patients with CSU, evaluate their quality and usability, and assess the technological preferences and needs of both patients and physicians. Based on these insights, recommendations for the development of future patient-centered CSU apps were derived.

## Methods

### Ethical Considerations

This analysis was approved by the Ethics Committee of the University of Würzburg (reference: 20230918 02) in accordance with the ethical standards established by the Declaration of Helsinki. All participants gave informed consent. Data was fully anonymized; no personal information was collected. No compensation was provided to the participants.

The reporting of this study followed established principles for transparent and structured reporting of research: STROBE (Strengthening the Reporting of Observational Studies in Epidemiology) guidelines (Checklist 2).

### App Screening

Between January 1 and March 31, 2024, 2 independent dermatologists with expertise in the management of urticaria conducted a systematic search of the Apple App Store, Google Play Store, and the internet using search engines (eg, Google and Safari) for mobile apps designed for patients with CU. Search terms used were “urticaria,” “spontaneous urticaria,” “hives,” “itching,” “angioedema,” “wheal,” and “swelling.” Inclusion criteria were defined as (1) availability on at least 1 of the specified platforms (Apple App Store, Google Play Store, or internet-based) and (2) language support in either German or English. Exclusion criteria comprised the (1) presence of advertisements, (2) a lack of patient-centered content—meaning content that primarily focuses on supporting the care and self-management of patients, or (3) a primary focus on clinical trials or scientific conferences. Apps containing advertisements were excluded in order to avoid potential disruptions to user experience and to control for confounding effects on usability [33]. Each app was assessed individually and independently. Discrepancies between reviewers were resolved through discussion. No automation tools were used during the screening process.

The primary outcomes for which data were sought included app functionality (eg, symptom tracking, disease activity scores, and medication reminders), user engagement features (eg, personalization and notifications), and data protection or privacy measures. All available features and descriptions provided in app store listings or within the apps themselves were reviewed. All data reflected the app status at the time of assessment.

For each app, the following variables were collected, if available: app name, platform availability (Apple App Store, Google Play Store, Google, and Safari), search term used, app topic (primary focus or intended purpose), total number of hits per search term, supported languages (German and English), app store rating and number of user ratings, target user group (patients and health care professionals), cost (free or paid), presence of advertisements, date of access, and inclusion of specific features such as symptom scores or disease monitoring tools.

A total of 15 apps were identified. After applying the inclusion and exclusion criteria, only one app, the CRUSE Control app, met the required criteria. The app was available on both Apple App Store and Google Play Store and supported German and English languages.

This study focuses on comparing app quality and usability between patients and physicians, rather than providing absolute ratings of individual apps.

### Raters’ Characteristics

Patients were randomly selected from our outpatient clinic to reduce selection bias and obtain a diverse user perspective. Similarly, physicians were randomly selected among those working in the same outpatient clinics and regularly treating patients with CSU. As the primary aim of the study is to assess app quality and usability, detailed information on participants’ social background, educational level, and clinical characteristics (eg, comorbidities or urticaria subtype) was not collected. This decision was made to minimize participant burden and maintain focus on the usability aspects of the app within the scope of this exploratory investigation.

Patients and physicians tested the CRUSE Control app version corresponding to the operating system available on their personal smartphones (iOS or Android). If they did not own a mobile device, they used a provided iPhone.

### Evaluation of App Quality

The Mobile Application Rating Scale (MARS) is a widely used tool for evaluating the quality of mobile apps [34]. It consists of 19 items across four categories: engagement, functionality, aesthetics, and information. Each item is rated on a 5-point Likert scale, with higher scores indicating higher quality. The overall MARS score is calculated as the mean value of these four subscale scores. In addition, there is a separate “subjective” section to capture the user’s personal impression of the app.

For this study, the validated German version of Mobile Application Rating Scale (MARS-G) [35] and the end-user version of Mobile Application Rating Scale (uMARS) were applied to assess the quality of MHAs. The uMARS represents a simplified version of the MARS designed for end users and has been shown to reliably assess the quality of MHAs [17]. The MARS-G includes an additional section, which evaluates therapeutic quality and safety across 4 dimensions: patient benefit, therapist benefit, potential risks, and transferability to routine care. It is referred to as the Psychotherapy Section in the questionnaire provided by Messner et al [35].

Although the German translation of the uMARS used in this study has not been formally published, its items are identical to those of the validated MARS-G. A comparative analysis between physicians’ and patients’ MARS and uMARS was conducted.

For clarity, the term “MARS” or “uMARS” is used throughout the manuscript to refer to this German version.

### Evaluation of App Usability

The G-MAUQ (German mHealth App Usability Questionnaire), the German validated version of the MAUQ, is a usability assessment tool for MHAs [36]. With 18 questions in 3 subcategories, it assesses the user’s perspective of the app’s ease of use, interface, and overall satisfaction and usefulness.

A total of 23 physicians assessed the app, appropriate for patients with CSU, using MARS-G to evaluate quality and G-MAUQ to assess usability. Simultaneously, 16 patients with CSU rated the same app, applying the uMARS for quality assessment and the G-MAUQ for usability evaluation. Prior to completing the evaluations, all participants were instructed to test each app for at least 10 minutes. A comparative analysis between physicians’ and patients’ G-MAUQ was conducted.

### Evaluation of Technical Affinity

The physicians’ and patients’ technology affinity was assessed using the affinity for technology interaction (ATI) scale and the Mobile Device Proficiency Questionnaire (MDPQ-16). The ATI is a well-established and widely used measure of an individual’s general affinity for technology consisting of 9 questions [37]. The 16-question MDPQ-16 is also used to assess technology affinity specifically related to the use of mobile devices [38].

Spearman rank correlation coefficient and Pearson correlation coefficient were used to correlate the ATI scale and MDPQ-16 score with ages of physicians and patients and the MDPQ-16. A comparative analysis between physicians’ and patients’ ATI and MDPQ-16 was conducted.

### Statistical Analysis

The calculations were performed using SPSS, version 23 (IBM Deutschland GmbH). Following the testing of the data for normal distribution with Shapiro-Wilk, either the Mann-Whitney *U* or *t* test was used. *P* values less than .05 were considered statistically significant. Correlation analysis was conducted using both the Spearman and Pearson correlation coefficients.

### Evaluation of Patients’ Need for a Patient App

Both physicians and patients completed a questionnaire specifically designed for this analysis. This questionnaire included open-ended questions regarding MHAs and their needs and expectations regarding such apps.

## Results

### App Screening and Selection

**Figure 1.**
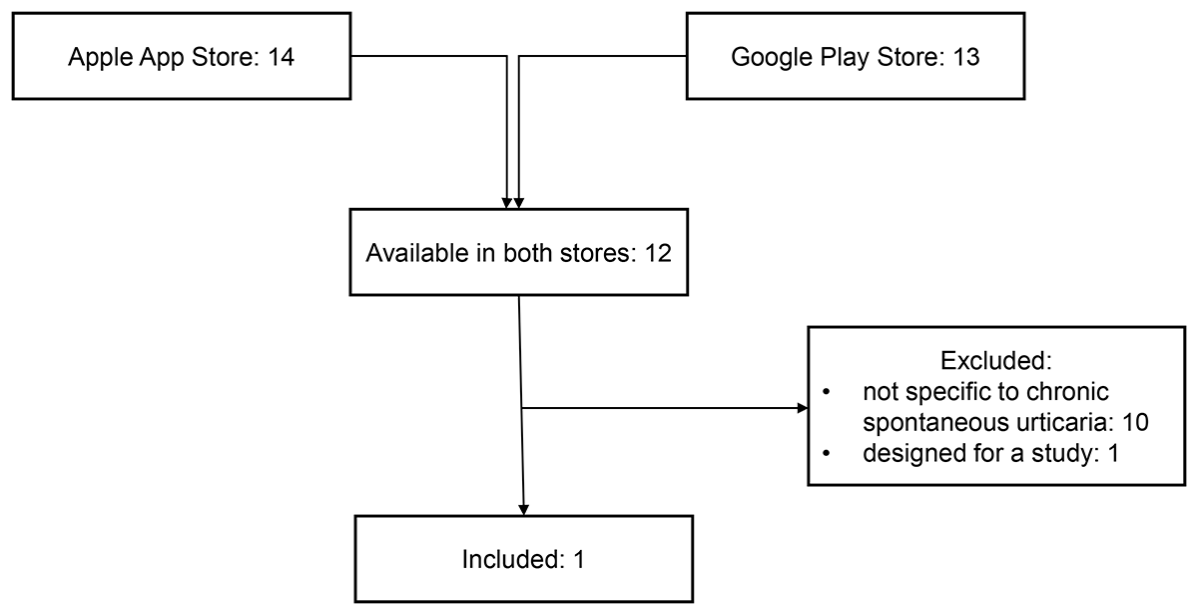
App selection.

A total of 15 urticaria-related apps were identified: 14 in the Apple App Store and 13 in the Google Play Store. Of these, 12 were identified in both App Stores, and 11 of these apps were excluded for the following reasons: not specific to CSU (n=10) and not patient-centered (n=1; Figure 1).

Only 1 currently available patient-centered urticaria app was identified: the CRUSE Control Urticaria app, a CU self-evaluation app available in both Apple App Store and Google Play Store. It was developed by Urticaria Centers of Reference and Excellence and is available in English and German. We tested version 1.0.69, last updated in November 2023.

The app offers patients with CU the opportunity to receive support in managing their condition. It provides a platform for daily documentation of symptoms and impact on quality of life within a few minutes. By sharing data with the treating physician, the efficacy of the current therapy and treatment can be optimized.

The app comprises 6 principal sections, each of which provides distinct functionality to assist patients with CU. In the “Daily Documentation” section, patients are required to evaluate the impact of urticaria over the preceding 24 hours. The questions are based on established scales, including the UAS and the Angioedema Activity Score. Additionally, patients are queried on a monthly basis regarding disease management, with questions based on the UCT and the Angioedema Control Test. The impact on the patient’s quality of life is assessed using the Chronic Urticaria Quality of Life Questionnaire and the Angioedema Quality of Life Questionnaire. Furthermore, daily medication intake and dosage are recorded.

The “Results Review” section enables users to monitor their daily progress in relation to the UAS and Angioedema Activity Scores, as well as their long-term disease management via the UCT, Angioedema Control Test, and quality of life indicators. In the “Profile View” section, patients may view and update their personal information. The “Preparation for Doctor’s Visit” section allows patients to prepare relevant information and questions for their upcoming appointment. Furthermore, patients have the option of uploading images of their urticaria in this section. A reminder function is provided to ensure that important appointments and medication dosages are not overlooked. Ultimately, the “Information about Urticaria” section offers valuable, patient-oriented material to facilitate comprehension of the condition. The link to the “Hello Professor” function provides valuable medical information that expands patients’ knowledge and increases their confidence in dealing with the disease.

The data collected can be summarized in a report for the physician, which may be transmitted via email or shared through a QR code. The integration of these elements facilitates uninterrupted and comprehensive observation of the condition, while also enhancing communication between patients and health care professionals (Table 1).

**Table 1.** Overview of patient-centered features in the CRUSE Control app.

Section/feature	Description/functionality	Notes/relevance to patient care
“Daily Documentation”	Daily symptom tracking (UAS^a^, AAS^b^), monthly disease management (UCT^c^, AECT^d^), quality of life (CU-Q2oL^e^, AE-QoL^f^), medication intake	Enables monitoring of disease activity and therapy efficacy
“Results review”	Visualization of progress and long-term disease control	Supports self-management and gives insight into therapy outcomes
“Profile View”	View and update personal information	Personalization of patient data
“Preparation for Doctor’s visit”	Upload images, prepare questions, reminders for appointments and medication	Enhances communication with physician and appointment readiness
“Information about Urticaria”	Educational content about the condition, “Hello Professor” cross-reference	Improves patient understanding and confidence
“Data Sharing”	Generate report for physician via email or QR code	Facilitates continuous monitoring and physician feedback

a UAS: urticaria activity score.

b AAS: angioedema activity score.

c UCT: urticaria control test.

d AECT: angioedema control test.

e CU-Q2oL: Chronic Urticaria Quality of Life Questionnaire.

f AE-QoL: Angioedema Quality of Life Questionnaire.

### Raters’ Characteristics

The app was evaluated by 23 dermatologists and 16 patients with CSU. Mean age was 34.0 (SD 9.7) years in physicians and 40.0 (SD 13.4) years in patients. The physicians’ group was comprised of 6 men and 17 women, while the patients’ group included 6 men and 10 women. All patients experienced multiple episodes of urticaria per month, lasting at least 12 weeks in total. Additionally, they reported sleep disorders and anxiety related to their condition.

### Evaluation of App Quality, Including Comparative Analysis of Patients’ and Physicians’ Data

Only the CRUSE Control app met the inclusion criteria and was included in this analysis. The results of the MARS and uMARS scores for the app are shown in Tables2  3.

**Table 2.** MARS^a^ score of the CRUSE Control app assessed by 23 physicians. Maximum MARS score: 5 points. In addition to the validated German version of Mobile Application Rating Scale core sections, the table includes the psychotherapy section—an extension in the German MARS that evaluates therapeutic quality across 4 dimensions (patient benefit, therapist benefit, risks, and transferability to routine care) as described by Messner et al [35].

	CRUSE Control app**,** mean (SD)
MARS score	4.03 (0.45)
Engagement score	3.67 (0.71)
Functionality score	4.47 (0.55)
Esthetics score	4.10 (0.56)
Information score	3.86 (0.49)
Subjective quality	3.49 (0.42)
Psychotherapy score	3.83 (0.61)

a MARS: Mobile Application Rating Scale.

**Table 3.** uMARS^a^ score of the CRUSE Control app assessed by 16 patients with urticaria. Maximum uMARS score: 5 points.

	CRUSE Control app, mean (SD)
uMARS score	4.06 (0.40)
Engagement score	3.55 (0.62)
Functionality score	4.75 (0.41)
Esthetics score	4.10 (0.45)
Information score	3.82 (0.81)
Subjective quality	3.35 (0.60)

a uMARS: end-user version of Mobile Application Rating Scale.

The quality of the CRUSE Control app was evaluated by both physicians and patients using the MARS or its user version (uMARS). Physicians and patients provided similar ratings, with an average score of 4.03 (SD 0.45) out of 5 for physicians and 4.06 (SD 0.40) for patients (*P*=.83), suggesting an overall high quality of the app. In most subcategories, including engagement (*P*=.58), esthetics (*P*=.97), information (*P*=.86), and subjective quality (*P*=.64), the ratings were closely aligned between the 2 groups. However, patients rated the functionality of the app significantly higher than physicians, with an average score of 4.75 (SD 0.41) compared to 4.47 (SD 0.55; *P*=.04). The subcategory measuring “Psychotherapy” was only included in the MARS for physicians. This section addresses the quality criteria for apps with respect to patient safety, the use of the app’s presentation, and the quality of the therapeutic offerings. They scored it with an average of 3.83 (SD 0.61).

### Evaluation of App Usability, Including Comparative Analysis of Patients’ and Physicians’ Data

The usability of the CRUSE Control app was assessed similarly by both physicians and patients. Using the G-MAUQ to measure usability, physicians gave an average score of 5.85 (SD 0.71) out of 7, while patients rated it slightly lower with an average score of 5.76 (SD 0.41) out of 7. No significant difference was found between the 2 groups (*P*=.64), as shown in Table 4. In the subcategories of G-MAUQ, both physicians and patients rated “Ease of Use” (6.27, SD 0.68 vs 6.45, SD 0.57), “Interface and Satisfaction” (6.04, SD 0.68 vs 6.00, SD 0.52), and “Usefulness” (5.30, SD 0.98 vs 4.91, SD 0.87) similarly. However, when looking at the absolute scores, patients rated “Usefulness” 0.39 points lower than physicians, though this difference was not statistically significant (*P*=.20).

**Table 4.** Evaluation of the CRUSE Control app with G-MAUQ^a^ and G-MAUQ subscales by physicians and patients. Maximum G-MAUQ score: 7 points.

	Physicians (n=23), mean (SD)	Patients (n=16), mean (SD)	*P* value
G-MAUQ	5.85 (0.71)	5.76 (0.41)	.68
Ease of use	6.27 (0.68)	6.45 (0.57)	.51
Interface and satisfaction	6.04 (0.68)	6.00 (0.52)	.83
Usefulness	5.30 (0.98)	4.91 (0.87)	.20

a G-MAUQ: German mHealth App Usability Questionnaire.

### Evaluation of Technical Affinity, Including Comparative Analysis of Patients’ and Physicians’ Data

The ATI score and the MDPQ-16 score, which evaluate the users’ affinity for technology, were recorded for both patients and physicians.

Physicians achieved a mean ATI score of 3.50 (SD 0.66) out of 6 points and a MDPQ-16 score of 4.81 (SD 0.26) out of 5 points. Patients, on the other hand, had a mean ATI score of 4.00 (SD 0.88) and a MDPQ-16 score of 4.74 (SD 0.45; *P*=.05). Although there were no statistically significant differences between the 2 groups, both groups considered themselves highly experienced in mobile device usage, as indicated by their similar MDPQ-16 scores (physicians: 4.81, SD 0.26; patients: 4.74, SD 0.45; *P*=.60), as shown in Table 5.

**Table 5.** Evaluation of technical affinity using the ATI^a^ scale and short version of MDPQ-16^b^ score. Maximum affinity for technology interaction score: 6 points; maximum MDPQ-16 score: 5 points.

	Physicians (n=23), mean (SD)	Patients (n=16), mean (SD)	*P* value
ATI	3.50 (0.66)	4.00 (0.88)	.05
MDPQ-16	4.81 (0.26)	4.74 (0.45)	.60

a ATI: affinity for technology interaction.

b MDPQ-16: Mobile Device Proficiency Questionnaire.

The Spearman correlation coefficient revealed a slight negative correlation between the ATI score and age for both physicians (−0.224; *P*=.30) and patients (−0.290; *P*=.28). The younger the physicians and patients were, the more likely they were to consider themselves technology-savvy (ATI).

A small positive correlation was observed between the ATI score and MDPQ-16 score, with a coefficient of 0.256 (*P*=.24) for physicians and 0.322 (*P*=.22) for patients.

When analyzing both groups together, the Spearman correlation between ATI score and age was −0.206 (*P*=.21), and between ATI score and MDPQ-16 score, it was 0.309 (*P*=.06).

### Patients’ Expectations and Comments

The open-ended questionnaire provided valuable insights into patients’ expectations and preferences regarding MHAs for managing urticaria. The 16 patients expressed a high level of openness to using MHAs to support their condition. Key functionalities that patients found particularly important included symptom tracking (n=14, 88%), photo documentation (n=15, 94%), medication management (n=14, 88%), and a note-taking and question feature (n=14, 88%). Almost half of the patients indicated a desire for an introductory guide to the app and technology training, as well as a digital companion to assist with navigation and provide encouragement. Additionally, 10 (63%) patients expressed interest in having a chat function with their doctor and a platform to connect with other individuals affected by urticaria.

A majority of the 16 patients (n=11, 69%) also requested a feature that would help organize and remind them of appointments. Other specific areas where patients sought support included information and educational content (n=11, 69%), tips for symptom management (n=11, 69%), psychosomatic support (n=5, 32%), and holistic treatment approaches (n=10, 63%).

To ensure regular use of the app, patients indicated that it would need to offer tangible improvements in their care, particularly by helping them manage their condition more effectively. Under these conditions, patients were open to using the app more than three times a week.

Additional patient wishes included “disease progression documentation,” “symptom tracking,” “support during acute flare-ups,” “therapy suggestions,” “features for identifying triggers,” “emergency support features,” “behavioral guidance for daily living,” and “user-friendly interface.”

Regarding the design of the CRUSE Control app, 14 (88%) out of 16 patients rated it at less than or equal to 7 out of 10 points. The information content was rated favorably by 12 (75%) out of 16 patients (≥7/10 points), while the usability and functionality were also rated positively by 12 (75%) and 10 (63%), respectively.

## Discussion

### Principal Findings

This study provides the first systematic analysis of MHAs for patients with CSU available in the App Stores and online. The patient cohort (mean age 40, SD 13.4 y; 6 men and 10 women) reflected the typical demographic pattern in CSU, with women being affected approximately twice as often as men [5,7,8]. The physician cohort (mean age 34, SD 9.7 y; 6 men and 17 women) represented the younger and predominantly female demographic commonly observed among university hospital staff in dermatology [39]. Despite broad inclusion criteria, only 1 out of 15 identified CSU apps met all predefined requirements. This highlights a substantial gap in the digital support landscape for CSU and underscores the need for targeted research and development of high-quality, evidence-based apps.

The episodic nature of CSU presents significant challenges for an accurate assessment of disease severity and activity [2]. Physicians rarely have the opportunity to observe patients during active flare-ups and rely on retrospective patient reports, photographs, and time-consuming interviews, which may result in misclassification and suboptimal treatment decisions. However, current guidelines recommend regular monitoring, ideally daily to weekly, using validated instruments such as the UAS and UCT to guide therapeutic adjustments [15]. MHAs have the potential to provide the solution. They constitute promising tools for continuous real-time symptom documentation, consolidating data into overviews to support both patients and physicians, and enhancing patient self-management and confidence. As their use in health care is becoming increasingly popular and steadily expanding, there is a growing need for their comprehensive and standardized evaluation [40]. A systematic review and evaluation of available medical apps, as provided by this study, using established and validated evaluation tools, is essential to gather feedback from both users (patients) and physicians (prescribers). This feedback will play a critical role in the ongoing development of MHAs [24,41]. The more closely an MHA is aligned with the needs of its users, the greater the likelihood of sustained engagement and long-term use [42].

The identified app, the CRUSE Control app, received high ratings across all MARS subdomains, including engagement, functionality, esthetics and information, with mean ratings of 4.03 (SD 0.45) on the MARS and 4.06 (SD 0.40) on the uMARS. However, high-quality ratings alone do not necessarily ensure sustained use [43]. Research has consistently shown that many MHAs, even those with favorable initial evaluations, experience a sharp decline in user engagement over time. In a mental health app, for instance, retention rates dropped to as low as 3.3% after 30 days [44]. Long-term adherence poses a major challenge in digital health [45] but is essential for achieving meaningful clinical outcomes. This is particularly important for chronic conditions such as CSU, which require ongoing monitoring of symptoms.

The positive evaluation of the CRUSE Control app is promising. Its high functionality and intuitive design facilitates rapid symptom documentation. This can reduce time burden and potentially support sustained user engagement, a factor consistently recognized as essential for long-term adherence in previous studies [46,47].

Although a user-friendly design may enhance initial engagement, sustained engagement ultimately depends on the app’s ability to meet the specific needs of its target users [47]. In this study, the differing priorities of consumers were reflected in the evaluations.

Physicians rated the functionality of the CRUSE Control app significantly lower than patients (*P*=.04) [41,48]. They rather emphasized clinical relevance, accuracy, reliability, and integration into workflows. Their focus was on precise data collection, treatment support, and evidence-based decision-making to ensure that the app meets medical standards and functions without errors. Clinical quality was also confirmed by the physicians’ rating of the psychotherapy subcategory (mean 3.83, SD 0.61). It was relatively high, indicating favorable assessments of patients’ safety, the suitability of app-based delivery, and the quality of therapeutic content.

In contrast, patients valued ease of use, intuitive navigation, and practical features for daily self-management. Rapid symptom documentation, completed within minutes, was seen as a major advantage. It reduced user burden and likely promoted adherence and sustained engagement. In subcategory functionality, patients appreciated particularly its usability and its role in everyday health management.

The G-MAUQ usability score reflects this positive perception of both groups. Physicians and patients alike rated the CRUSE Control app highly for usability, with “ease of use” and “user satisfaction” scores nearing 6 out of 7 on the G-MAUQ. The ratings of the app’s “usefulness” were slightly lower, though the differences between the groups were not significant.

Despite differences in priorities and usability needs, these high ratings indicate that both groups find significant value in the app: physicians for its clinical use and patients for its support in self-management.

Both patients with CSU and physicians demonstrated high technological affinity and competence in using mobile devices, as reflected by high ATI and MDPQ-16 scores. Hence, MHAs can be adopted and used effectively by this group [49,50]. A slight negative correlation between age and technological affinity was observed. This indicates that younger individuals tend to be more comfortable with digital tools. Older users may need additional support to ensure equitable access and sustained engagement.

Patient feedback in the open-ended questionnaire demonstrated a clear willingness to use MHAs for managing CSU, particularly if the app offered real benefits such as symptom tracking, photo documentation, and medication management. Patients wish for psychosocial support, education, and communication features. A future app could integrate community and peer support functionalities through health care professional–moderated forums or support groups, aiming to reduce patient isolation and encourage sharing personal experiences.

### Limitations

Data collection focused on app ratings, without detailed clinical data such as disease severity or duration, which limits potential correlations with app use. The short-term evaluation of a single app without direct comparison to other CSU apps may have introduced some bias. The relatively young study population also limits the generalizability of the findings to older patients.

A major limitation of this study is that only 1 app met the predefined inclusion criteria at the time of the search, potentially introducing selection bias. The findings primarily reflect the characteristics of this single app and should be interpreted with caution regarding the broader CSU mHealth landscape. Because CRUSE Control is a well-developed and medically guided app, there is a possibility of performance or reporting bias, which could have led to an optimistic assessment of the potential of mHealth for CSU.

Future studies should include broader clinical data, longer follow-up, and direct comparisons across multiple CSU apps to reduce bias and enhance generalizability.

### Conclusions

Despite broad search criteria, only 1 app met all inclusion requirements, highlighting a significant gap in digital support for patients with CSU. Both patients and physicians rated the app highly in terms of quality, usability, and information content. Patients rated functionality significantly higher, reflecting differing priorities between ease of daily use and clinical rigor. High technological affinity among both groups suggests favorable conditions for MHA adoption. Patients expressed clear interest in features supporting symptom tracking, medication management, and psychosocial support, emphasizing the need for patient-centered designs and onboarding assistance. Overall, MHAs show great potential to enhance CSU care.

While these findings are limited to a single app, they indicate that MHAs may have the potential to enhance CSU care. Future research should incorporate broader clinical parameters, longer follow-up, and diverse patient populations to further assess app effectiveness and optimize digital health tools for CSU management.

## Supplementary material

10.2196/71435 Checklist 1 PRISMA checklist.

10.2196/71435 Checklist 2 STROBE checklist.

## Abbreviations

DiGA: Digital Health Applications/ Digitale Gesundheitsanwendung
ATI: affinity for technology interaction
CSU: chronic spontaneous urticaria
CU: chronic urticaria
DiGA: digitale gesundheitsanwendung (English: digital health apps)
G-MAUQ: German mHealth App Usability Questionnaire
MARS: Mobile Application Rating Scale
MDPQ-16: Mobile Device Proficiency Questionnaire
MHA: mobile health app
PRISMA: Preferred Reporting Items for Systematic reviews and Meta-Analyses
STROBE: Strengthening the Reporting of Observational Studies in Epidemiology
UAS: urticaria activity score
UCT: urticaria control test
uMARS: end-user version of Mobile Application Rating Scale
